# The Blessings and Benefits of the Massachusetts Medical Society

**Published:** 1847-01

**Authors:** 


*The Blessings and Benefits of the Massachusetts Medical Society.* By a Member.—Permit me, through your very useful Journal, to inquire whether any patent has been obtained for the *modus operandi* of reconciling the principles and avowed objects of the Massachusetts Medical Society, with the recent practice of some of its leading members. Probably but few country practitioners are acquainted with the process, and we presume they would be willing to pay for the secret, not only because any man, unless he be one of the veriest numskulls in the world, must readily perceive that all future discoveries in medicine, especially if they be important ones, may be patented under the auspices of a part of the members of the M. M. Society, and the secret sold to another part, but also because the inventor must be a man of very surprising genius.

We would suggest that as opium and other narcotic remedies administered for the prevention or relief of pain, whether it be taken into the stomach, injected into the rectum, rubbed upon the skin, or inhaled into the lungs, have no direct tendency to cure disease, except in cases where they prevent the shock of a surgical operation, or save the powers of life from being frittered away by continued agony (in which case it must be agreed on all hands they work even curative effects); and also as vaccination from the prevention of small-pox has no direct tendency to cure disease; and moreover, as the discovery of a remedy for the prevention of phthisis would have no tendency to cure disease; so also it seems naturally to follow that as the reconciling of the aforsaid principles with the aforsaid practice would have no tendency to cure disease, no objection ought to be made against its being patented.

By the way, we would inquire whether as new patents come out for new discoveries in medicine, the country practitioners may take turns with the city practitioners in writing the puffs and paying for the patent.

Finally, we might suggest to the members of the Massachusetts Medical Society the propriety of exercising all due meekness and quietness while the patenting business is going on, and of submitting to everything to which they are obliged to submit.—*Ibid.*

